# High Protective Efficacy of Probiotics and Rice Bran against Human Norovirus Infection and Diarrhea in Gnotobiotic Pigs

**DOI:** 10.3389/fmicb.2016.01699

**Published:** 2016-11-02

**Authors:** Shaohua Lei, Ashwin Ramesh, Erica Twitchell, Ke Wen, Tammy Bui, Mariah Weiss, Xingdong Yang, Jacob Kocher, Guohua Li, Ernawati Giri-Rachman, Nguyen Van Trang, Xi Jiang, Elizabeth P. Ryan, Lijuan Yuan

**Affiliations:** ^1^Department of Biomedical Sciences and Pathobiology, Virginia-Maryland College of Veterinary Medicine, Virginia Tech, BlacksburgVA, USA; ^2^School of Life Science and Technology, Institut Teknologi, BandungWest Java, Indonesia; ^3^National Institute of Hygiene and EpidemiologyHanoi, Vietnam; ^4^Division of Infectious Diseases, Cincinnati Children’s Hospital Medical Center, CincinnatiOH, USA; ^5^Department of Environmental and Radiological Health Sciences, College of Veterinary Medicine and Biomedical Sciences, Colorado State University, Fort CollinsCO, USA

**Keywords:** probiotics, rice bran, human norovirus, diarrhea, gnotobiotic pigs

## Abstract

Probiotics have been recognized as vaccine adjuvants and therapeutic agents to treat acute gastroenteritis in children. We previously showed that rice bran (RB) reduced human rotavirus diarrhea in gnotobiotic pigs. Human noroviruses (HuNoVs) are the major pathogens causing non-bacterial acute gastroenteritis worldwide. In this study, *Lactobacillus rhamnosus* GG (LGG) and *Escherichia coli* Nissle 1917 (EcN) were first screened for their ability to bind HuNoV P particles and virions derived from clinical samples containing HuNoV genotype GII.3 and GII.4, then the effects of LGG+EcN and RB on HuNoV infection and diarrhea were investigated using the gnotobiotic pig model. While LGG+EcN colonization inhibited HuNoV shedding, probiotic cocktail regimens in which RB feeding started 7 days prior to or 1 day after viral inoculation in the LGG+EcN colonized gnotobiotic pigs exhibited high protection against HuNoV diarrhea and shedding, characterized by significantly reduced incidence (89 versus 20%) and shorter mean duration of diarrhea (2.2 versus 0.2 days), as well as shorter mean duration of virus shedding (3.2 versus 1.0 days). In both probiotic cocktail groups, the diarrhea reduction rates were 78% compared with the control group, and diarrhea severity was reduced as demonstrated by the significantly lower cumulative fecal scores. The high protective efficacy of the probiotic cocktail regimens was attributed to stimulation of IFN-γ^+^ T cell responses, increased production of intestinal IgA and IgG, and maintenance of healthy intestinal morphology (manifested as longer villi compared with the control group). Therefore, probiotic cocktail regimens containing LGG+EcN and RB may represent highly efficacious strategies to prevent and treat HuNoV gastroenteritis, and potentially other human enteric pathogens.

## Introduction

Human noroviruses, non-enveloped viruses with a positive-strand RNA genome, are the major pathogens causing non-bacterial acute gastroenteritis worldwide (Pringle et al., 2015). In the United States, HuNoVs have replaced HRVs as the single most common cause of viral gastroenteritis in children and adults (Bresee et al., 2012; Payne et al., 2013). After approximately 1.2 days of incubation (Lee et al., 2013), HuNoV gastroenteritis generally lasts for 2–3 days and consists of nausea, vomiting, and diarrhea (Lopman et al., 2004). More severe and prolonged illness can occur in specific risk groups, including infants, the elderly, and immunocompromised patients (Murata et al., 2007; Hall et al., 2012; Green, 2014). Given the tremendous disease burden and economic loss associated with HuNoVs infection (Patel et al., 2008; Ahmed et al., 2014), vaccines and therapeutics are in great demand to prevent and treat these infections. However, due to the lack of a robust culturing system and a suitable small-animal model, HuNoVs vaccine development and antiviral research have long been hampered. Promising vaccines have focused on recombinant capsid proteins, including VLPs and P particles (Kocher and Yuan, 2015). Appropriate animal models are essential tools to facilitate investigation of vaccine candidates and therapeutic strategies. Neonatal gnotobiotic (Gn) pigs recapitulate the pathologic hallmarks of enteric viral infection and associated immune responses in the gastrointestinal tract of young children (Yang and Yuan, 2014). Currently, as the only animal model that supports the oral route of HuNoV infection, develops diarrhea, and sheds virus in feces, Gn pigs are used to evaluate viral pathogenesis and vaccine efficacy with high translational validity to humans (Cheetham et al., 2006; Bui et al., 2013; Kocher et al., 2014).

Probiotic bacteria are increasingly recognized as vaccine adjuvants and therapeutic agents to treat acute gastroenteritis in children (Licciardi and Tang, 2011; Schnadower et al., 2015). The potential mechanisms include competing with pathogens for nutrients and colonization sites, producing antimicrobial metabolites, enhancing protective immune responses, and reducing intestinal permeability (Ng et al., 2009). Notably, Gram-positive probiotics *Lactobacillus* spp. have been extensively evaluated for their beneficial effects against viral infection and diseases. These include reducing HRV and vesicular stomatitis virus infection in cell cultures (Botic et al., 2007; Maragkoudakis et al., 2010) and promoting HRV-specific immune responses, which contribute to shortened HRV-induced diarrhea in animal models (Zhang et al., 2008; Wen et al., 2014, 2015) and human clinical trials (Guandalini et al., 2000; Sindhu et al., 2014; Szajewska et al., 2014). Gram-negative EcN is also a well-characterized probiotic used to treat diarrhea in infants and young children (Henker et al., 2007, 2008), as well as in neonatal large animals (von Buenau et al., 2005; Schroeder et al., 2006). The beneficial health effects are mediated via improving intestinal barrier function (Hering et al., 2014) or moderating inflammatory responses (Splichalova et al., 2011), which could protect Gn piglets from lethal infection of *Salmonella* Typhimurium (Splichalova et al., 2011). In addition, EcN was recently shown to have HRV-binding and immunomodulatory properties, resulting in significantly reduced HRV infection and diarrhea in Gn pigs (Kandasamy et al., 2016). Probiotics can act as adsorbents for HuNoV P particles, and the presence of *L. casei* BL23 and EcN might inhibit P particle attachment to epithelial cells (Rubio-del-Campo et al., 2014). *Enterobacter cloacae* (EC) is a commensal bacterium that can bind to HuNoV by surface HBGA and inhibit HuNoV infectivity in Gn pigs (Miura et al., 2013; Lei et al., 2016b). Taken together, diarrhea-reducing probiotics may inhibit HuNoV infectivity *in vivo*, most likely by the binding between bacteria and virions.

Rice bran, an underutilized by-product of rice milling, contains a variety of prebiotic and bioactive components that modulate gut microbiota and potentially prevent chronic diseases, including diabetes, cancer, metabolic syndrome, and cardiovascular disease (Sheflin et al., 2015). In mouse studies, dietary RB feeding increased production of fecal and serum IgA (Henderson et al., 2012), and RB glycoproteins ameliorated cyclophosphamide-induced immunosuppression by restoring splenic lymphocytes (Park et al., 2016), indicating that RB promoted the development of mucosal and systemic adaptive immunity. Chemically engineered RB glucans possessed anti-cytomegalovirus activity by blocking viral entry of target cells (Ray et al., 2013). Recently, therapeutic effects of RB in inhibiting enteric infections and reducing diarrhea have been gaining attention. In a clinical trial, Biobran (modified arabinoxylan RB) improved irritable bowel syndrome symptoms, presumably resulting from its anti-inflammatory and/or immunomodulatory effects (Kamiya et al., 2014). In our previous Gn pig studies, dietary RB feeding significantly enhanced HRV vaccine immunogenicity and reduced HRV-induced diarrhea (Yang et al., 2014). RB could also protect against HRV diarrhea in the presence of probiotics by preventing intestinal epithelial damage and promoting innate immune responses (Yang et al., 2015). Therefore, its beneficial effects on gastrointestinal health support RB as a promising agent against HuNoV infection.

In this study, aiming to develop an effective and ready-to-use anti-HuNoV therapeutic strategy, we first screened a group of probiotics to identify the virus-binding bacteria using HuNoV P particles and native virions. Subsequently, probiotics and RB were evaluated individually or combined as cocktail regimens for their effects on HuNoV infection and diseases in the well-established Gn pig model (Bui et al., 2013). Finally, the mechanisms of antiviral and diarrhea-reducing activities from those treatments were explored.

## Materials and Methods

### Viruses and Bacteria

A human stool sample containing the HuNoV GII.4/2006b variant 092895 (GenBank KC990829) was collected in 2008 at the Cincinnati Children’s Hospital Medical Center from a child with norovirus gastroenteritis. The sample pool was processed as an oral inoculum for HuNoV infection studies in Gn pigs (Bui et al., 2013). A human stool sample containing the HuNoV GII.3/20110200 (GenBank KX355506) was collected in 2011 at the Thai Binh Pediatric Hospital (Thai Binh province, Vietnam) from a female child with norovirus gastroenteritis. *L. reuteri* (ATCC 23272), *L. acidophilus* (strain NCFM), *L. rhamnosus* GG (ATCC 53103), and *L. bulgaricus* (ATCC 11842) were cultured in lactobacilli MRS broth (Neogen Corporation) anaerobically using BBL^TM^ GasPak^TM^ jar system with Anaerobe Sachets (BD) under static condition at 37°C. EcN (a gift from Dr. Jun Sun, Rush University, Chicago, IL, USA) and *Enterobacter cloacae* (ATCC 13047) were cultured in Luria Bertani medium at 37°C and in nutrient broth at 30°C, respectively, with shaking at 250 rpm.

### Purification of HuNoVs and VP1 Sequencing

The pooled human stools containing HuNoVs were diluted 10-fold with diluent #5 (Minimal Essential Medium with 1% penicillin-streptomycin and 1% HEPES) and mixed thoroughly with an equal volume of Vertrel XF (Miller-Stephenson), and viruses were purified by CsCl gradient centrifugation as described previously (Guix et al., 2007). VP1 of GII.4/2006b variant 092895 was cloned and sequenced previously (Kocher et al., 2014). GII.3/20110200 viral RNA was extracted from the purified virus by TRIzol LS and reverse transcribed by SuperScript III Reverse Transcriptase (Thermo Fisher Scientific) using universal GII.3 reverse primer 5′-TAG CCC CTG CAT TAA CTA-3′ and following the manufacturer’s instructions. The GII.3 VP1 was cloned by a nested PCR with primer set 1 (forward: 5′-TGA GCA CGT GGG AGG GCG-3′ and reverse: 5′-TAG CCC CTG CAT TAA CTA-3′) and primer set 2 (forward: 5′-CAC CAT GAA GAT GGC GTC GAA T-3′ and reverse: 5′-TTA TTG AAT CCT TCT ACG CC-3′) into pENTR directional TOPO vector (Thermo Fisher Scientific). The GII.3 VP1 fragment in the recombinant plasmids were sequenced by Virginia Bioinformatics Institute at Virginia Tech, and the predominant sequence was used for the preparation of P particles.

### P Particles and Transmission Electron Microscopy

The region coding for the P domain was amplified from the recombinant plasmids containing VP1 capsid gene of HuNoV GII.3 or GII.4 as described above. The P domains were cloned into prokaryotic expression vector pET21a (EMD Millipore) as previously described (Rubio-del-Campo et al., 2014). A 6×His-Tag was incorporated to the N-terminus of P proteins by forward primers, and a cysteine-rich peptide CDCRGDCFC was incorporated to the C-terminus of P proteins by reverse primers to enhance the P particle stability (Tan and Jiang, 2005). P proteins were expressed in *E. coli* strain BL21 (New England Biolabs) and purified via HisPur Ni-NTA Spin Columns (Thermo Fisher Scientific) following the manufacturer’s instructions. Protein production was monitored by SDS-PAGE and InVision His-tag In-gel Stain (Thermo Fisher Scientific). Protein concentrations were measured spectroscopically by Quick Start^TM^ Bradford protein assay (Bio-Rad). Electron microscopy formvar carbon square grids (Electron Microscopy Sciences) were pretreated with 1% aqueous Alcian blue for 5 min. After washes, P particles of genotype GII.3 or GII.4 were diluted in PBS to 5 μg/ml and absorbed to the grids for 1 min. The grids were stained with 3% phosphotungstic acid pH 7.0 for 1 min and viewed with a JEOL JEM 1400 transmission electron microscopy.

### Binding of P Particles and Virions to Bacteria

After the initial inoculation into fresh culture medium, bacteria were grown overnight and sub-cultured at 1:50 for 2–3 h until OD_600_ reached 0.4–1.0, which was the log phase of growth. Bacteria were washed three times and resuspended with PBS to an OD_600_ of 1.0. Then 10 μg P particles or 10^6^ viral genome copies of purified HuNoVs were incubated with 1 ml bacteria for 1 h at 37°C, then the mixture was centrifuged and washed three times with PBS. To measure the remaining P particles attached to bacteria, the bacterial pellets were resuspended with 100 μl Laemmli sample buffer (Bio-Rad) and boiled for 10 min, and 20 μl of sample was loaded to 10% SDS-PAGE gel and analyzed by Western Blot using HRP conjugated anti-His-Tag antibody (MA1-21315-HRP, Thermo Fisher Scientific). To measure the remaining virions, the total RNA of the bacterial pellets was extracted by 750 μl TRIzol LS, and HuNoV genomes were detected by a one-step TaqMan qRT-PCR with primers targeting all GII viruses (Kageyama et al., 2003; Lei et al., 2016a). For controls, *Enterobacter cloacae* were heat-killed at 65°C for 40 min, then blocked with 5 μl of A antigen antibody (sc-69951, Santa Cruz) and H antigen antibody (sc-52369, Santa Cruz) at 37°C for 20 min before adding P particles or virions.

### Gnotobiotic Pigs and Treatment Groups

Near-term Yorkshire cross-breed pigs were derived via hysterectomy by veterinarians and maintained in sterile isolator units as described previously (Meyer et al., 1964). Neonatal Gn pigs (male and female) were randomly assigned to the five treatment groups upon derivation: cocktail-7d (*n* = 5), cocktail+1d (*n* = 5), RB-7d (*n* = 4), LGG+EcN (*n* = 5), control (*n* = 9). To initiate the colonization of LGG and EcN, 10^4^ CFU of each were mixed in five ml of Minimal Essential Medium and administered orally to pigs on PPD 3, 5, and 7. The low dosage was chosen on purpose to be well below the therapeutic practice (10^9^ to 10^12^ CFU). LGG and EcN fecal shedding were determined by rectal swab sampling of pig feces and enumeration of colonies grown on media agar plates as described previously (Yang et al., 2015). For RB feeding of pigs, heat-stabilized and gamma-irradiated RB (Calrose variety) was added to pigs’ milk diet by replacing 10% daily calorie intake (Yang et al., 2015). Daily feeding started 7 days prior to or 1 day after HuNoV inoculation until euthanasia. All pigs were orally inoculated on PPD33 with 6.43 × 10^5^ viral genome copies of HuNoV GII.4/2006b variant 092895. To reduce gastric acidity, four ml 200 mM sodium bicarbonate were given to pigs 15 min prior to inoculation. Fecal consistency and virus shedding were assessed daily until euthanasia on PPD40 where blood, tissues, and intestinal contents were collected. Fecal consistency scores were obtained based on previous scaling system (Bui et al., 2013), and fecal virus shedding was measured by a one-step TaqMan qRT-PCR as described previously (Lei et al., 2016a).

### Flow Cytometry Analysis

Mononuclear cells (MNCs) were isolated from the duodenum, ileum, spleen, and blood as described previously (Yuan et al., 2008). 2 × 10^6^ of MNCs were restimulated *in vitro* with P particle (12 μg/ml for the spleen and 6 μg/ml for others), positive control PHA (10 μg/ml), or mock control in E-RPMI media for 17 h at 37°C. Brefeldin A (B6542, Sigma-Aldrich, 5 μg/ml) and anti-CD49d monoclonal antibody (561892, BD Biosciences, 1 μl/ml) were added at 12 h post-incubation to block the secretion of cytokines and enhance the stimulation, respectively. IFN-γ^+^ CD4^+^ and CD8^+^ T cells were quantified by flow cytometry as described previously (Yuan et al., 2008). Isotype matched irrelevant antibodies were included as negative gate controls. Mock-stimulated samples indicate the total IFN-γ^+^ T cells, while the increased cell populations of P particle-stimulated over mock-stimulated samples indicate HuNoV-specific IFN-γ^+^ T cells.

### ELISA for Total Immunoglobulin and IFN-γ

The total immunoglobulin (Ig) titers in intestinal contents were determined by ELISA as described previously (Lei et al., 2016a). Intestinal IFN-γ titers were measured by Swine IFN-γ VetSet^TM^ ELISA development Kit (Kingfisher Biotech) following the manufacturer’s instructions.

### Jejunum Histopathology

Jejunum tissue was collected after pig euthanasia, fixed in 4% paraformaldehyde for 12–16 h, paraffin embedded, sectioned into 5 μm slices and placed on positively charged slides, for routine H&E staining. A pathologist who was blinded to the sample identifications evaluated the villus length using an ocular micrometer under a light microscope.

### Ethical Statement

Stool collection protocols were approved by the Institutional Review Boards of the Cincinnati Children’s Hospital Medical Center (IRB#: 2008-1131) and the National Institute of Hygiene and Epidemiology – Vietnam (IRB#: 15-IRB), written consent was provided by parents or guardians of the children. Animal experimental protocols were approved by the Institutional Animal Care and Use Committee at Virginia Tech (IACUC protocol: 13-187-CVM and 14-108-CVM). All sample collection and experimental procedures were conducted in accordance with the approved guidelines.

### Statistics

Statistics were performed using GraphPad Prism 6.0 (GraphPad Software) with analyses indicated in table notes and figure legends. Statistical significance was determined at the level of *P* < 0.05.

## Results

### Probiotic Bacteria Bind to HuNoVs

**FIGURE 1:**
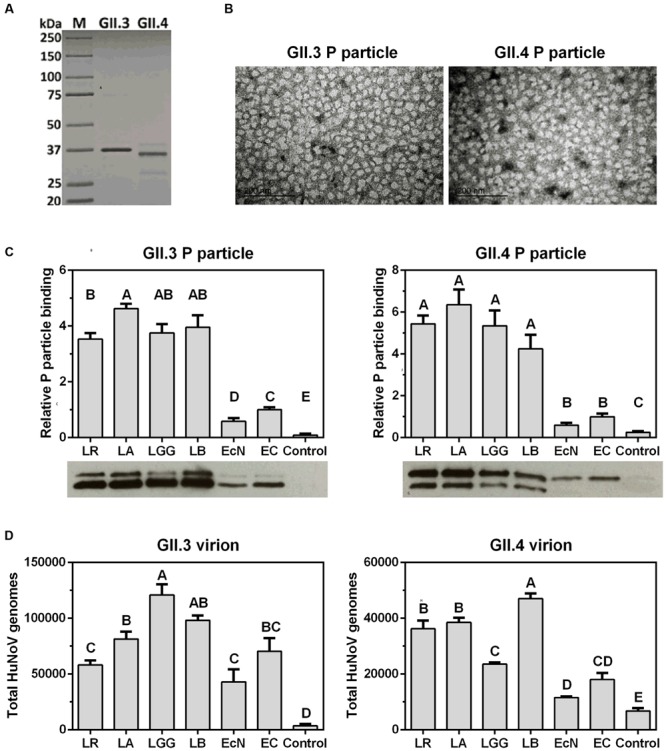
**Probiotic bacteria bind to HuNoV P particles and virions. (A)** SDS-PAGE analysis of the 6×His tagged P proteins expressed and purified from the prokaryotic system. Lane 1, protein standard marker. Lanes 2 and 3, GII.3 and GII.4 P proteins. **(B)** Transmission electron micrographs of P particles. Scale bar, 200 nm. **(C)** Probiotic bacteria bind to P particles. Bottom panels, representative Western Blot using anti-His-Tag antibody showing the P particles bound to bacteria. Top panels, four independent experiments were analyzed by Image J. The relative bindings are referred to that of EC. **(D)** Probiotic bacteria bind to virions. The virions bound to bacteria were quantified by qRT-PCR for viral genomes. The experiments were repeated three times independently. Controls were heat-killed and A&H antigen antibodies-blocked EC. Data are presented as mean ± SEM. Statistics was determined by one-way analysis of variance (ANOVA). Different letters indicate significant differences among groups (*P* < 0.05), while shared letters indicate no significant difference.

Although HuNoV genotype GII.4 accounts for the most global acute gastroenteritis outbreaks (Pringle et al., 2015), GII.3 is emerging and becoming predominant in some underdeveloped areas (Liu et al., 2014; Van Trang et al., 2014; Ayouni et al., 2015). To explore the interactions between HuNoVs and probiotics, we first cloned the capsid VP1 genes of GII.3 and GII.4 from clinical stool samples, then the P-domains were cloned and proteins were expressed with N-terminal 6×His-Tag to facilitate their detection and purification (**Figure 1A**). The formation of P particles was not compromised as indicated by a negative staining electron microscopy (**Figure 1B**).

P particles were first used as a model to determine HuNoVs interactions with probiotics, including a Gram-negative strain EcN and four Gram-positive lactobacilli strains, i.e., *L. reuteri* (LR), *L. acidophilus* (LA), *L. rhamnosus* GG (LGG), and *L. bulgaricus* (LB). Since EC can bind to HuNoVs specifically by surface HBGA (Miura et al., 2013), native EC was used as a positive control and HBGA A&H antibodies-blocked EC was a negative control in the binding assays. After incubation of P particles and bacteria, the P particles remaining on the bacterial surface were quantified by Western Blot using anti-His-Tag antibody. The results showed that all the tested bacteria were able to bind to both GII.3 and GII.4 P particles, and lactobacilli strains had significantly higher binding capacity than those of EcN and EC (**Figure 1C**). Additionally, LA was stronger than LR in binding to GII.3 P particle, while EcN was weaker than EC. The four lactobacilli strains did not differ from each other in binding to GII.4 P particle, and neither did EcN and EC (**Figure 1C**). Similarly, the binding assays were performed using HuNoV virions purified from stool samples. Unlike the P particles, GII.3 virions had comparable binding to all tested bacteria except for the higher binding to LGG, whereas GII.4 virions shared the binding pattern with P particle except for the lower binding to LGG and higher binding to LB (**Figure 1D**). These data suggest that probiotic bacteria can bind to HuNoVs.

### LGG+EcN Inhibited HuNoV Shedding and RB Reduced Diarrhea in Gn Pigs

**FIGURE 2:**
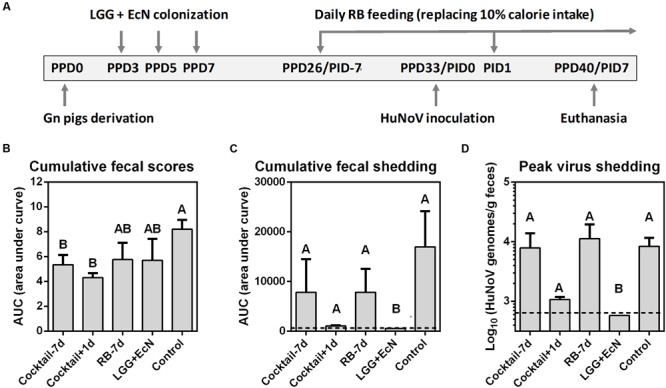
**Design and summary of Gn pig study. (A)** Experimental timeline. PPD, post-partum day; PID, post-inoculation day. Cumulative fecal scores **(B)** and shedding **(C)** are shown as area under curve from daily measurements of individual pigs (Supplementary Figure S1). **(D)** Mean peak virus shedding titers from PID1 to PID7 in individual pigs. Sample sizes are shown in **Table 1**. Data are presented as mean ± SEM. Dashed lines indicate limit of detection. Statistics were determined by Kruskal–Wallis test. Different letters indicate significant differences among groups (*P* < 0.05), while shared letters indicate no significant difference.

To develop a ready-to-use anti-HuNoV therapeutic strategy, LGG and EcN were chosen for the evaluation of their potential antiviral effects in the Gn pig model of HuNoV infection and diarrhea, since they could bind HuNoVs *in vitro* and are commercially available as diarrhea-reducing probiotics. Previous study showed that RB protected against HRV-induced diarrhea in the presence of LGG and EcN (Yang et al., 2015), we tested RB feeding and/or LGG+EcN co-colonization in five treatment groups in this study: cocktail-7d (*n* = 5), pigs were pre-colonized with LGG and EcN, RB feeding started 7 days prior to HuNoV inoculation; cocktail+1d (*n* = 5), pigs were pre-colonized with LGG and EcN, RB feeding started 1 day after virus inoculation; RB-7d (*n* = 4), RB feeding started 7 days prior to inoculation; LGG+EcN (*n* = 5), pigs were colonized with LGG and EcN only; Control (*n* = 9), non-RB fed and non-LGG+EcN colonized. All pigs were inoculated with a HuNoV GII.4/2006b variant 092895 on PPD33/PID0 and euthanized on PID7 (**Figure 2A**).

Fecal consistency and virus shedding were assessed daily after the HuNoV inoculation (Supplementary Figure S1). The results summarized in **Table 1** showed that compared to the control group, LGG+EcN group had similar rates of HuNoV diarrhea (89 versus 60%), yet undetectable HuNoV shedding. RB-7d group had a slightly shorter mean duration of diarrhea (2.2 versus 1.3 days) and significantly delayed shedding onset (2.8 versus 6.3 days). More importantly, cocktail-7d and cocktail+1d groups had a significantly lower incidence (20%), delayed onset (3.9 versus 7.0 and 7.2 days, respectively), shorter mean duration of diarrhea (2.2 versus 0.2 days), and shorter mean duration of virus shedding (3.2 versus 1.0 days). In both cocktail groups, the diarrhea reduction rates were 78% [1 – (% of treated pigs with diarrhea/% of control pigs with diarrhea)], and the reduced severity of diarrhea was also shown by the significantly lower cumulative fecal scores (**Figure 2B**). Interestingly, only the LGG+EcN group had significantly reduced cumulative and peak virus shedding compared to the control group. RB feeding with or without LGG+EcN colonization did not significantly alter virus shedding pattern, except that shedding in the cocktail+1d group trended lower when compared to the other RB fed groups and the controls (**Figures 2C,D**).

**Table 1:** Incidence of diarrhea and fecal virus shedding in Gn pigs after HuNoV GII.4 challenge^a^.

Group	*n*	Diarrhea^b^			Virus shedding^b^		
		Pigs with diarrhea (%)^∗^	Mean days to onset (SEM)^∗∗^	Mean duration days (SEM)^∗∗^	Pigs shedding virus (%)^∗^	Mean days to onset (SEM)^∗∗^	Mean duration days (SEM)^∗∗^
Cocktail-7d	5	1 (20%)^A^	7.0 (1.0)^A^	0.2 (0.2)^A^	4 (80%)^A^	4.0 (1.2)^AB^	1.0 (0.3)^AB^
Cocktail+1d	5	1 (20%)^A^	7.2 (0.8)^A^	0.2 (0.2)^A^	5 (100%)^A^	4.8 (0.2)^AB^	1.0 (0)^A^
RB-7d	4	3 (75%)^AB^	3.3 (1.6)^AB^	1.3 (0.5)^AB^	3 (75%)^A^	6.3 (0.8)^A^	1.5 (0.6)^AB^
LGG+EcN	5	3 (60%)^AB^	4.0 (1.6)^AB^	1.8 (1.1)^AB^	0^B^	8.0 (0)^C^	0^C^
Control	9	8 (89%)^B^	3.9 (0.7)^B^	2.2 (0.4)^B^	8 (89%)^A^	2.8 (0.8)^B^	3.2 (0.9)^B^

*^a^Gn pigs were inoculated with a HuNoV GII.4 2006b variant 092895 at 33 days of age. Diarrhea and virus shedding were monitored by daily rectal swab sampling and RT-qPCR after inoculation. Calculation included all pigs in each group from PID1 to PID7.*

*^b^If diarrhea or virus shedding was not observed, the days to onset was recorded as 8 and the duration days was recorded as 0 for statistical purposes.*

*^∗^Fisher’s exact test or ^∗∗^Kruskal–Wallis test was used for comparisons. Different letters indicate significant differences among treatment groups (*P* < 0.05), while shared letters indicate no significant difference.*

### RB Promoted the Colonization of EcN but not LGG in Gn Pigs

**FIGURE 3:**
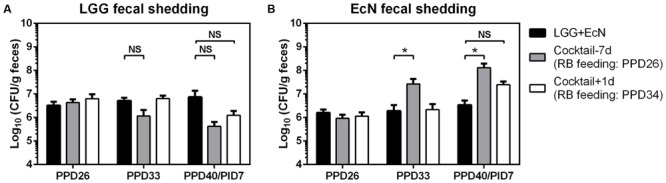
**LGG and EcN fecal shedding.** Pig feces were collected by rectal swab and suspended in PBS. The concentrations of LGG **(A)** and EcN **(B)** were determined in serial dilution of fecal samples and enumeration of colony forming unit (CFU) grown on MRS or LB media agar plates. Sample sizes are shown in **Table 1**. Data are presented as mean ± SEM. Statistics were determined by Kruskal–Wallis test. NS, not significant, ^∗^*P* < 0.05.

The colonization of LGG and EcN in Gn pigs was confirmed by their fecal shedding on PPD26 (**Figures 3A,B**). After the beginning of RB feeding on PPD26 in the cocktail-7d group and on PPD34 in the cocktail-1d group, LGG fecal shedding appeared to decrease in both groups, however, statistical significance was not observed for these differences (**Figure 3A**). On the other hand, RB feeding significantly increased EcN fecal shedding in the cocktail-7d group and slightly in the cocktail-1d group (**Figure 3B**). Taken together, these results indicate the differential effects of RB on the co-colonization of probiotic bacteria.

### LGG+EcN and RB Stimulated the Production of IFN-γ^+^ T Cells

**FIGURE 4:**
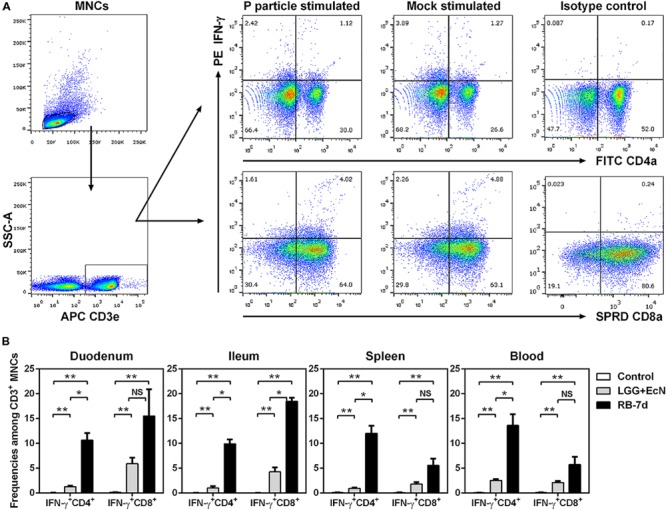
**LGG+EcN and RB stimulated IFN-γ^+^ T cell responses. (A)** Gating strategies for IFN-γ^+^ CD3^+^CD4^+^ (Th) cells and CD3^+^CD8^+^ (CTL) cells. Representative dot plots showing frequencies of HuNoV-specific (P particle stimulated) and non-specific (mock stimulated) IFN-γ^+^ T cells in ileum isolated from LGG+EcN colonized Gn pigs. SSC-A, side scatter area; APC, allophycocyanin; FITC, fluorescein isothiocyanate; SPRD, spectral red; PE, phycoerythrin. **(B)** Non-specific IFN-γ^+^ T cells in intestinal (duodenum, ileum) and systemic (spleen, blood) tissues on PID7. Sample sizes are shown in **Table 1**. Data are presented as means ± SEM. Statistics were determined by Kruskal–Wallis test. NS, not significant, ^∗^*P* < 0.05, ^∗∗^*P* < 0.01.

To elucidate the mechanisms of the inhibitory effects of LGG+EcN and RB on HuNoV infection and diarrhea, their immunomodulatory roles were first assessed regarding effector T cells. After euthanasia on PID7, MNCs were isolated from both intestinal and systemic lymphoid tissues, and the frequencies of IFN-γ^+^ CD4^+^ and CD8^+^ T cells were determined by flow cytometry (**Figure 4A**). MNCs were stimulated with P particle to detect HuNoV-specific IFN-γ^+^ T cells, which was the increased frequency compared to the mock stimulated sample. For pigs in the control, LGG+EcN, and RB-7d groups, no significant increase of IFN-γ^+^ T cells was observed in P particle stimulated MNCs (**Figure 4A** and data not shown), suggesting low or short-term HuNoV-specific IFN-γ^+^ T cell responses. However, compared with control pigs, both LGG+EcN colonization and RB feeding significantly increased frequencies of non-specific total IFN-γ^+^ T cells (**Figure 4B**). In addition, compared with the LGG+EcN group, the RB-7 group had significantly higher frequencies of IFN-γ^+^ CD8^+^ T cell population in ileum and IFN-γ^+^ CD4^+^ T cell population in all assayed lymphoid tissues (duodenum, ileum, spleen, and blood; **Figure 4B**), indicating that RB has strong stimulatory effects on total IFN-γ^+^ T cell responses, which may contribute to the reduction of HuNoV diarrhea in Gn pigs.

### Probiotics plus RB Cocktail Regimens Enhanced Gut Immunity

**FIGURE 5:**
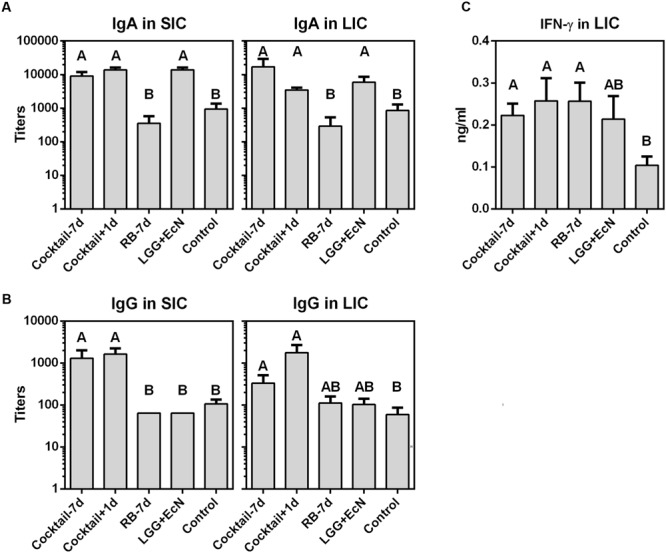
**IgA, IgG, and IFN-γ levels in intestinal contents after HuNoV infection.** Total IgA **(A)** and IgG **(B)** titers in small and large intestinal contents (SIC and LIC) were measured by ELISA. **(C)** IFN-**γ** concentration in LIC was measured by ELISA. Sample sizes are shown in **Table 1**. Data are presented as mean ± SEM. Statistics were determined by Kruskal–Wallis test. Different letters indicate significant differences among groups (*P* < 0.05), while shared letters indicate no significant difference.

The immunomodulatory roles of LGG+EcN and RB on gut immunity were evaluated by testing total intestinal IgA, IgG, and IFN-γ levels, since PID 7 is too early to detect virus-specific IgA and IgG antibody responses. Compared with the control group, the cocktail-7d, cocktail+1d, and LGG+EcN groups had significantly higher IgA titers in both small and large intestinal contents (SIC and LIC), but the increase was not observed in the RB-7d group (**Figure 5A**), indicating that LGG+EcN but not RB enhanced the production of IgA. The cocktail-7d and cocktail+1d groups had significantly higher IgG titers in both SIC and LIC, whereas no differences were observed in either the LGG+EcN or RB groups (**Figure 5B**). Consistent with the strong stimulation of RB on total IFN-γ^+^ T cells (**Figure 4B**), significantly higher IFN-γ concentrations were detected in LIC from the cocktail-7d, cocktail+1d, and RB-7d groups (**Figure 5C**). In all, cocktail regimens remarkably enhanced gut immunity in Gn pigs by secretion of intestinal immunoglobulins and interferon, which might provide protection against HuNoV infection.

### Probiotics plus RB Cocktail Regimens Increased Jejunal Villi Length

**FIGURE 6:**
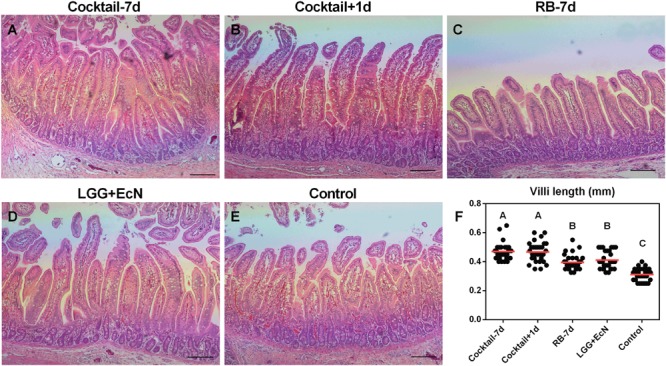
**LGG+EcN and RB are associated with longer villi. (A–E)** Representative images of H&E stained jejunum showing the villi length in the five groups. Scale bar, 0.25 mm. **(F)** 30 random villi including all pigs in each group were measured to quantify the villi length. Data are presented as means with individual points. Statistics were determined by Kruskal–Wallis test. Different letters indicate significant differences among groups (*P* < 0.05), while shared letters indicate no significant difference.

Villus blunting is a major manifestation of impaired intestinal health, such as in Crohn’s disease (Cadwell et al., 2010), celiac disease (Chand and Mihas, 2006), and virus-induced gastroenteritis (Hodges and Gill, 2010). To examine the beneficial effects of LGG+EcN and RB on the health of small intestine in Gn pigs, sections of jejunum were stained with H&E and evaluated for all the treatment groups after euthanasia. Compared with control, both LGG+EcN colonization and RB feeding were associated with significantly longer jejunal villus length. Their stimulatory roles might be additive as the two cocktail groups displayed greater villus length than either single treatment (**Figure 6**). These data indicate that the cocktail regimens promote the growth and health of intestinal epithelium, which might contribute to the protection of HuNoV-induced disease.

## Discussion

Robust cell culture and animal models have long been lacking for HuNoV propagation, as a result, clinical stool samples from patients are the only resource for HuNoV infection studies. P particles are promising surrogates as they exhibit surface conformation and receptor-binding profiles similar to the corresponding VLPs (Tan et al., 2008), and they have been validated as an *in vitro* model to evaluate viral binding with probiotics (Rubio-del-Campo et al., 2014). In this study, we first prepared HuNoV GII.3 and GII.4 P proteins, which displayed double bands as expected (**Figures 1A,C**; Rubio-del-Campo et al., 2014). The P particle structures were observed under electron microscopy. The binding assays with both P particles and native virions showed that their binding capacity with Gram-negative EcN was lower than that with Gram-positive lactobacilli, EcN was still included in this study due to its commercial availability, diarrhea-reducing properties on enteric pathogens such as HRV (Kandasamy et al., 2016), and potential inhibition of HuNoV attachment to epithelial cells (Rubio-del-Campo et al., 2014). It is likely that differential cell surface composition of Gram-negative and Gram-positive bacteria determines the observed differences, although surface components that are responsible for viral binding remain to be identified.

Bacterial microbiota was shown to facilitate persistent and acute MuNoV infection in mice (Jones et al., 2014; Baldridge et al., 2015), but the effects of different bacteria on MuNoV infectivity might vary as lactobacilli could inhibit MuNoV infection *in vitro* using RAW264.7 cell culture model and vitamin A inhibited MuNoV replication in mice by upregulating lactobacilli in gut microbiota (Lee and Ko, 2016). In this study, after HuNoV inoculation in Gn pigs colonized with LGG+EcN, virus fecal shedding was below the limit of detection, indicating significant inhibition on HuNoV infection by their colonization. Similar to the reduced virus shedding but unaffected incidence of diarrhea observed in EC colonized Gn pigs in the previous study (Lei et al., 2016b), LGG+EcN colonization did not alter the occurrence of diarrhea, suggesting that HuNoV gastroenteritis could be induced by extremely low viral loads and that anti-HuNoV agents inhibiting viral replication may have insufficient efficacy in reducing the disease. Given that bacterial anti-HuNoV capacity might depend on the extent of viral retention ability, it is likely that LGG plays a more important role on the inhibition of HuNoV infectivity than EcN, since LGG has a greater HuNoV-binding ability, but further investigations will be required to clarify the effects of LGG or EcN mono-colonization on HuNoV infection. Nevertheless, cocktail regimens containing LGG and EcN offer great promise to simultaneously protect against HuNoV and HRV infection (Kandasamy et al., 2016).

Although, RB was shown to promote the colonization of lactobacilli in mice (Henderson et al., 2012), LGG fecal shedding was lower after RB feeding in cocktail groups in this study. When colonized together with EcN in Gn pigs, LGG fecal shedding and concentration in intestinal tissues trended toward lower than those of single colonization (Kandasamy et al., 2016), suggesting that the presence of EcN might inhibit the growth of LGG. Thus, it is likely that higher growth of EcN led to lower growth of LGG after RB feeding, and underlying mechanisms utilized by EcN need to be identified, such as competing for the nutrients and colonization sites, improving intestinal barrier, and modulating immune responses (Splichalova et al., 2011; Hering et al., 2014). In all, higher protective efficacy against HuNoV shedding and diarrhea might be achieved only if RB and LGG are given.

Effector T cells are a crucial immune component to eliminate viral infected cells, and their responses in the small intestine are associated with protective immunity against HRV (Yuan et al., 2008). However, HuNoV infection or P particle vaccination did not significantly stimulate virus-specific IFN-γ^+^ CD4^+^ or CD8^+^ T cell responses (Kocher et al., 2014), and neither did LGG+EcN colonization nor RB feeding in this study. Still, significantly increased frequencies of non-specific IFN-γ^+^ T cells were observed especially after RB feeding, which might be correlated with the diarrhea-reducing property of RB, but a significant reduction in virus shedding was not observed along with the enhanced T cell responses in the RB-7d group, which was similar to our previous study on HRV (Yang et al., 2014). For the cocktail regimens, the intestinal IgA was increased by LGG+EcN alone, while the increased intestinal IgG might have been induced by the synergism between LGG+EcN and RB. The additive effects of the probiotics and RB appeared to be associated with longer jejunal villus length.

In this study, the cocktail-7d group displayed a 78% reduction of diarrhea, as well as significantly shortened duration of diarrhea and virus shedding after HuNoV challenge, indicating the regimen is an effective preventive measure. In addition, similar effects in reducing diarrhea and virus shedding were observed in the cocktail+1d group, in which RB feeding started 1 day after HuNoV challenge, thus this regimen could be considered as a therapeutic strategy to treat HuNoV gastroenteritis. The first HuNoV vaccine candidate evaluated in clinical trials was an adjuvanted monovalent GI.1 VLP, which provided 47 and 26% protection against Norwalk virus gastroenteritis and infection compared with the placebo group, respectively (Atmar et al., 2011). A bivalent VLP-based vaccine containing both GI.1 and GII.4 components is under development as well, and human clinical trials showed a 52% reduction in vomiting and/or diarrhea compared with the control after challenge (Debbink et al., 2014). Our previous evaluations of adjuvanted GII.4 VLP and P particle vaccines in Gn pigs demonstrated reductions of diarrhea by 60 and 47%, respectively (Kocher et al., 2014). Therefore, the probiotics plus RB cocktail regimens may provide an alternative strategy with better anti-HuNoV effects than the current vaccine candidates.

In summary, lactobacilli and EcN could bind to HuNoV P particles and virions derived from GII.3 and GII.4 clinical samples. Colonization with LGG+EcN completely inhibited HuNoV fecal shedding in Gn pigs. The two cocktail regimens had RB feeding started either 7 days prior to or 1 day after viral inoculation in the LGG+EcN colonized Gn pigs, and both regimens exhibited dramatic anti-HuNoV effects, including reduced incidence and shorter duration of diarrhea, as well as shorter duration of virus fecal shedding. The anti-HuNoV effects of the cocktail regimens were associated with the stimulated IFN-γ^+^ T cell responses, increased production of intestinal IgA and IgG, and longer villus length. Considering the natural source and commercial availability of probiotics and RB, the cocktail regimens may represent a novel, safe and ready-to-use strategy against diarrhea and infection caused by HuNoV infection and other enteric pathogens.

## Author Contributions

SL and LY conceived the project and designed the experiments. SL performed most experiments and analyzed data. AR, ET, KW, TB, MW, XY, JK, GL, and EG-R assisted with experiments. NVT, XJ, and EPR contributed key materials and/or reagents. SL and LY wrote the manuscript. All authors reviewed the manuscript before submission.

## Conflict of Interest Statement

The authors declare that the research was conducted in the absence of any commercial or financial relationships that could be construed as a potential conflict of interest.

## Abbreviations

EC: *Enterobacter cloacae;*
EcN: *Escherichia coli* Nissle 1917
Gn: gnotobiotic
HBGA: histo-blood group antigen
HRV: human rotavirus
HuNoV: human norovirus
LA: *Lactobacillus acidophilus;*
LB: *Lactobacillus bulgaricus;*
LGG: *Lactobacillus rhamnosus* GG
LR: *Lactobacillus reuteri;*
MuNoV: murine norovirus
PID: post-infection day
PPD: post-partum day
RB: rice bran
VLP: virus-like particle
