# Supplementary material for: High Protective Efficacy of Probiotics and Rice Bran against Human Norovirus Infection and Diarrhea in Gnotobiotic Pigs
Source: Front Microbiol. 2016 Nov 2;7:1699. doi: 10.3389/fmicb.2016.01699 (PMC5090003; doi:10.3389/fmicb.2016.01699)
Supplement: Supplementary file 1 [file Image_1.PDF]

Supplementary Material

High protective efficacy of probiotics and rice bran against human norovirus infection and diarrhea in gnotobiotic pigs

Shaohua Lei[1], Ashwin Ramesh[1], Erica Twitchell[1], Ke Wen[1], Tammy Bui[1], Mariah Weiss[1], Xingdong Yang[1], Jacob Kocher[1], Guohua Li[1], Ernawati Giri-Rachman[1,2], Nguyen Van Trang[3], Xi Jiang[4], Elizabeth P. Ryan[5], Lijuan Yuan[1,*]

[1]Department of Biomedical Sciences and Pathobiology, Virginia-Maryland College of Veterinary Medicine, Virginia Tech, Blacksburg, VA, USA.

[2]School of Life Science and Technology, Institut Teknologi, Bandung, West Java, Indonesia.

[3]National Institute of Hygiene and Epidemiology, Hanoi, Vietnam.

[4]Division of Infectious Diseases, Cincinnati Children's Hospital Medical Center, Cincinnati, OH, USA.

[5]Department of Environmental and Radiological Health Sciences, College of Veterinary Medicine and Biomedical Sciences, Colorado State University, Fort Collins, CO, USA.

***Correspondence:** Lijuan Yuan. E-mail: lyuan@vt.edu

Running title: Probiotics and rice bran against HuNoV

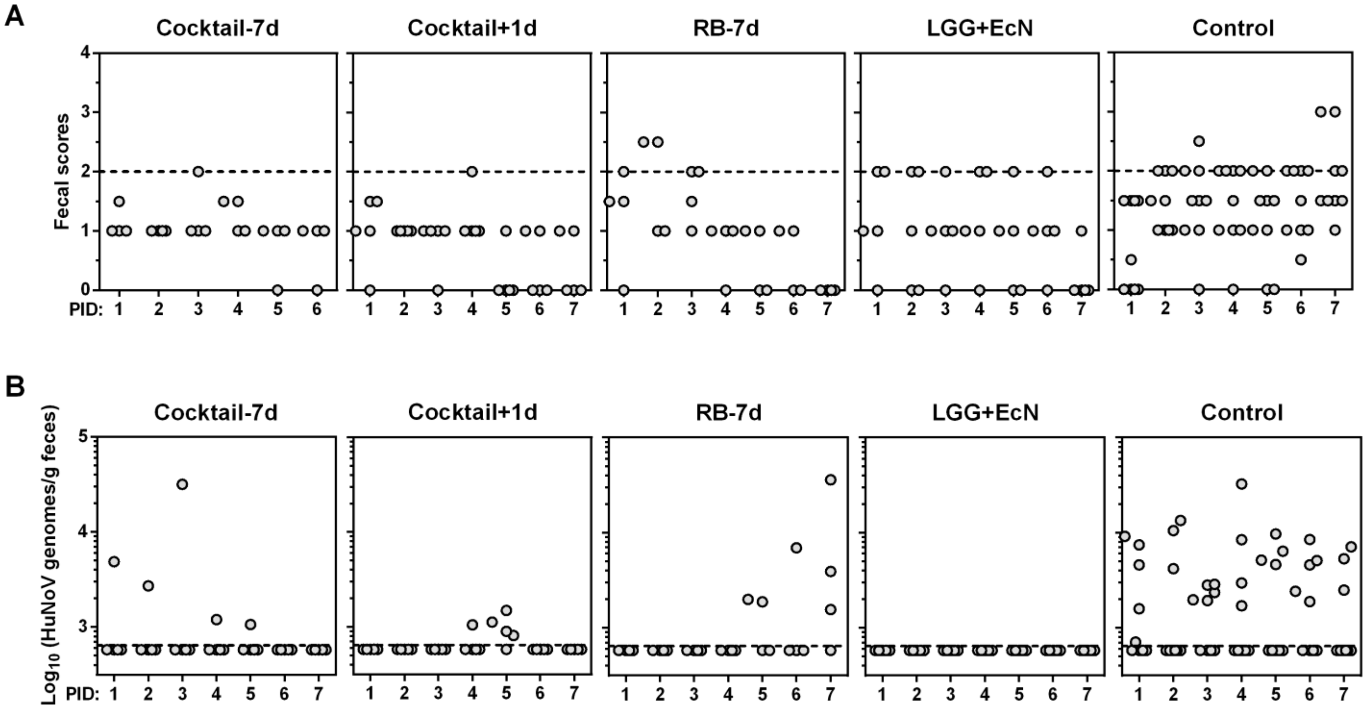

Supplementary Figure 1. Fecal consistency scores and HuNoV shedding. (**A**) Fecal consistency scores. Pig feces were collected by rectal swabs and scored as follows: 0, solid; 1, pasty; 2, semiliquid; and 3, liquid. Fecal scores of ≥2 as indicated by dashed lines were considered diarrheic. (**B**) Fecal HuNoV shedding. Viral titers in pig feces were measured by qRT-PCR, dashed lines indicate limit of detection. Data are presented as individual animal data points.
